# A Possible Correlation Between Auricular Angiomas and Breast Cancer Through Auricular Acupuncture Diagnosis: An Observational Case-Control Study

**DOI:** 10.7759/cureus.60834

**Published:** 2024-05-22

**Authors:** Antonello Lovato, Mario Biral, Marco Postiglione, Giuseppe Gagliardi, Veronica Gagliardi, Francesco Ceccherelli

**Affiliations:** 1 Integrative Medicine, Pain Therapy Clinic, Vicenza, ITA; 2 Complementary Medicine, A.I.R.A.S. (Italian Association for the Research and the Scientific Update), Padua, ITA; 3 Epidemiology and Public Health, A.I.R.A.S. (Italian Association for the Research and the Scientific Update), Padua, ITA; 4 Anesthesiology, AULSS 5 Polesana, Rovigo, ITA; 5 Pain Therapy, A.I.R.A.S. (Italian Association for the Research and the Scientific Update), Padua, ITA; 6 Medicine, University of Padua, Padua, ITA; 7 Anesthesiology and Pain Therapy, A.I.R.A.S. (Italian Association for the Research and the Scientific Update), Padua, ITA

**Keywords:** breast cancer, auricle angiomas, ear acupuncture diagnosis, auricular diagnosis, auricular acupuncture

## Abstract

Introduction

Auricular acupuncture (AA) can be used for both diagnosis and therapy. Diagnosis done with AA has become more prominent, with inspection by evaluating skin alterations considered the most important step. Literature on AA diagnosis in cancer patients is scarce. Globally, breast cancer (BC) is the most commonly diagnosed cancer in women.

Materials and methods

Subjects accessing the outpatient Breast Unit Clinic of Padua for BC were evaluated for auricle angiomas, with collected data including a number of angiomas, Romoli’s Sectogram sector of identified angiomas, laterality of the auricle with the angioma, age, and laterality of BC.

Results

Of the 438 subjects evaluated, 129 had BC, and 64 had an identifiable auricle angioma. The odds of an auricular angioma were higher in subjects with BC diagnosis, mainly localized in tumor area II and predominantly ipsilateral to the side affected by BC.

Conclusions

AA auricle inspection is a simple, quick, and easy diagnostic tool. Screening for the presence and location of auricular angiomas may help health practitioners refer women for BC screening for early diagnosis.

## Introduction

Diagnosis in auricular acupuncture (AA)

AA, unlike somatic acupuncture, is based on the concept of the auricle representing the whole body. With the term first suggested by Dale in 1974, AA is considered a micro-acupuncture system in traditional Chinese medicine (TCM) [1]. Similar to other microsystem methods, AA is characterized as having both diagnostic and therapeutic effects [2], with scientific evidence of diagnosis done with AA becoming increasingly available. In 1973, medical doctor S.T. Frank published a short article on the correlation between the presence of a particular auricular lobule crease or groove (i.e., Frank’s sign) and coronary artery disease (CAD) [3]. This correlation was recently confirmed in subjects with Frank’s sign on an auricle lobe, especially in bilateral cases, who showed higher rates of CAD after an angiographic study of their coronary arteries [4,5]. Oleson conducted an experiment on 40 subjects with chronic osteoarticular pain in which auricle tenderness elicited through the Pressure Pain Test (PPT) was assessed, and areas on the auricle with an elicitable different electrical skin resistance were identified through the Electrical Skin Resistance Test (ESRT). Based on the “active” areas on the auricle, Oleson was able to correctly determine the corresponding anatomical area of pain origin in 37 out of 40 subjects [6].

Inspection

In the TCM text “Nan Jing,” or “Yellow Emperor’s Classic of Eighty-One Difficult Issues,” written most likely during the Warring States period (426-221 BC), is found: “the skillful doctor knows by observation, the mediocre doctor by interrogation, the ordinary doctor by palpation” [7]. As such, in ancient times in China, it was thought that TCM physicians inspected the whole body, including the auricle. Following 1958, with the birth of auricular somatotopy, as proposed by Nogier, the Chinese AA School taught a two-step diagnosis method. The first step, known as the “Confirmation Step,” comprised formulating a diagnosis through the correlation between skin and cartilaginous alterations of the auricle and the auricular somatotopy as proposed by Nogier. The second step, known as the “Test Phase,” comprised auricle skin palpation to identify tender (i.e., active) points and, thus, identify areas and points that would receive AA treatment. Two prominent Western AA authors, Dr. Johan Nguyen and Dr. Marco Romoli, have investigated auricular diagnosis further. Nguyen described and classified the “lesion cutanee ponctuelle” (or spot-on skin lesion) according to nine categories: follicle, nevus, angioma, telangiectasia, comedone nodule, chondroplasia, scar, and hair hyperplasia [8]. Romoli is one of the authors who has been most involved in the study of auricular diagnosis, producing many scientific papers [9,10]. Romoli considered inspection to be the most important step of AA diagnosis. Together with the dermatologist Dr. Antonia Pata, Romoli proposed classifying skin alterations (SAs) based on their relation to changes in vascularization, pigmentation, keratinization, cartilage structure, skin structure, and sebaceous glands [11,12].

AA diagnosis in cancer patients

Although the literature on AA diagnosis among cancer patients is scarce, there are two scientific papers that positively correlate cancer with SAs. Zhu Dan described the concentration of SAs in two auricle areas (i.e., tumor areas I and II) on the helix tail, which correspond to Romoli’s Sectogram sectors 4-9 and 15-19, respectively, and identified a total of seven tumor areas, with the main ones being areas I and II. Dan then examined 115 subjects affected by cancer and 120 subjects affected by chronic non-neoplastic pathologies by means of ear inspection, finding that auricular SAs were more prevalent in the identified tumor areas among subjects affected by cancer [13]. Dr. Huang Li-Chun also described the presence of SAs in subjects affected by cancer, which were concentrated in tumor area II identified by Zhu Dan [14].

Breast cancer (BC)

Globally, BC is ranked the second most common site of cancer by incidence and the fourth most common site for cancer mortality for both sexes, and the most common site for incidence and mortality in women [15]. The incidence and prevalence of female BC are highest in Asia (42.9% and 39.0%, respectively) and Europe (24.4% and 28.2%, respectively). In 2020, over 2.3 million new cases of BC occurred globally, with broad geographic variability among the different regions of the world, with BC incidence reported to be 40 in 100,000 in some African and Asian countries, to over 80 in 100,000 in Australia, North America, and Europe [16]. BC incidence is highest in Western Europe and the U.S., and lowest in Africa and Asia, which could be attributed to vast underreporting of the disease, reflecting a falsely low prevalence in the latter regions [17]. Low BC incidence and high mortality rates have been observed in low- to middle-income countries, which have been associated with a lack of resources for preventative screening for early BC detection and adequate treatment resources [17,18].

In contrast, all European countries offer some form of BC screening. Nevertheless, there are considerable disparities across different geographical areas in Europe. It has been estimated that if all European countries achieved 100% coverage of biennial BC screening examinations in women aged 50-69 years, mortality due to BC would decrease by 23% in Eastern Europe, 21% in Western Europe, 15% in Southern Europe, and 9% [A1] [AL2] [A3] in Northern Europe [19]. According to a systematic review, most guidelines recommend screening mammography for women aged 40-74 years, with an optimal screening age range of 50-69 years [20]. Nevertheless, 9 out of 23 guidelines [A4] [AL5] included in the review recommend not setting an upper age limit for BC screening.

In contrast, all European countries offer some form of BC screening. Nevertheless, there are considerable disparities across different geographical areas in Europe. It has been estimated that if all European countries achieved 100% coverage of biennial BC screening examinations in women aged 50-69 years, mortality due to BC would decrease by 23% in Eastern Europe, 21% in Western Europe, 15% in Southern Europe, and 9% in Northern Europe [19]. According to a systematic review, most guidelines recommend screening mammography for women aged 40-74 years, with an optimal screening age range of 50-69 years [20]. Nevertheless, 9 out of 23 guidelines included in the review recommend not setting an upper age limit for BC screening.

Mammography is recommended as the primary screening modality for women at average risk of BC by all guidelines. Furthermore, guidelines suggest yearly, biennial, or even every three years as intervals for mammography screening [20]. Breast self-exam has been considered a controversial tool for the early detection of BC, which must not take the place of programmed screening mammograms and clinical breast exams. There has been a growing consensus acknowledging the need for global efforts to counteract the growing burden of BC, especially in transitioning countries where BC incidence is rising rapidly and mortality rates remain high.

## Materials and methods

Study design

**Figure 1:**
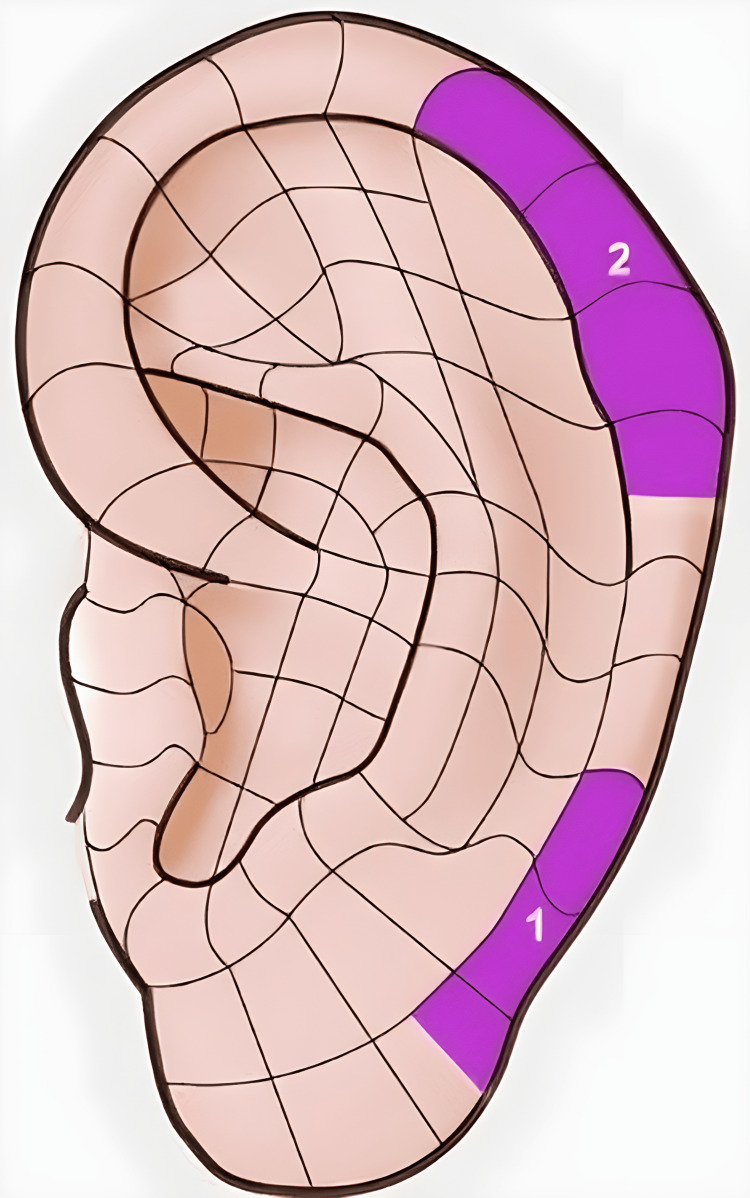
Tumor areas I and II on the auricle, as identified by Zhu Dan. Image was taken and modified from “Agopuntura Auricolare teoria e clinica”, Antonello Lovato, NOI Edizioni, 2019, with permission to publish.

**Figure 2:**
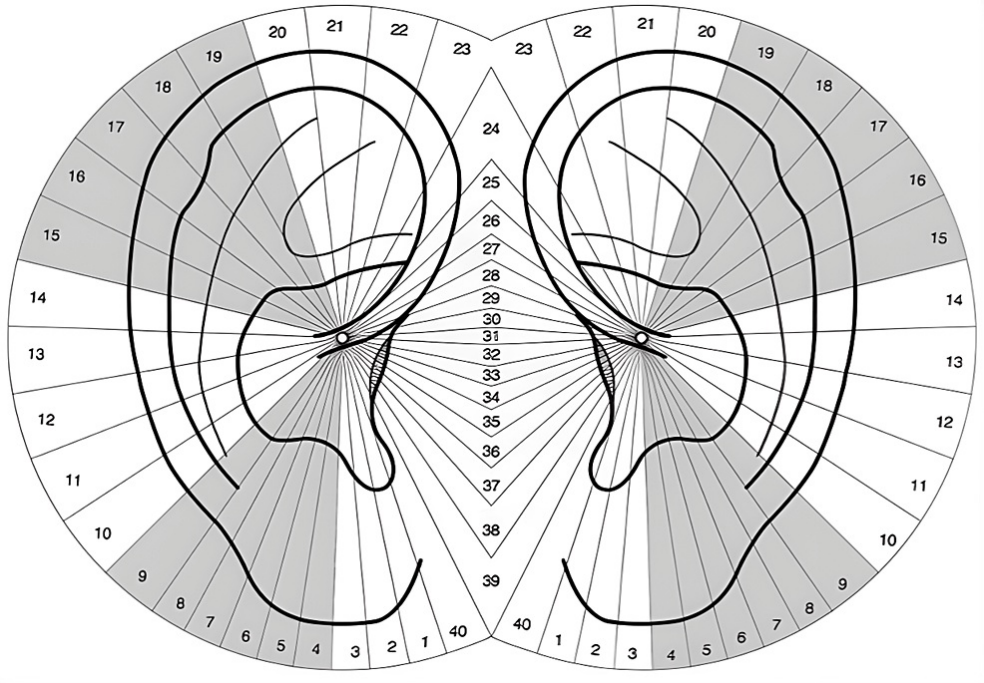
Romoli’s Sectogram. The sectors that correspond to the tumor areas I and II are shaded in grey. Image was taken and modified from “Agopuntura Auricolare teoria e clinica,” Antonello Lovato, NOI Edizioni, 2019, with permission to publish.

We conducted an observational case-control study to examine the correlation between the presence of an auricle angioma and BC. We also evaluated the locations of all auricle angiomas and determined the number of cases in tumor areas I or II (i.e., Romoli’s Sectogram sectors 4-9 or 15-19, respectively) (Figures 1-2). Finally, we assessed the relationship between the side of BC and the side of the auricle that presented the angioma [21].

Study population

Over a period of 12 months (i.e., January 15, 2017, to January 14, 2018), subjects who accessed the outpatient Breast Unit Clinic of Padua for first referral by their family doctor or General Practitioner (GP) (i.e., screening for BC early diagnosis) or for BC follow-up were enrolled in the study.

Study location

Subjects were referred to the outpatient Breast Unit Clinic from the catchment area of the Local Health Unit of Padua (i.e., Azienda Ulss 6 Euganea, Padova, Italy), with a catchment population of approximately 934,000 patients.

Selection criteria

The inclusion criteria for the study were as follows: female and ≥18 years of age. The exclusion criteria for the study were as follows: subjects already known by the examining doctor.

Data collection

Data were collected by a medical doctor on staff at the Breast Unit Clinic with certified knowledge of AA medicine (i.e., AA diagnosis and AA therapy). Data collection was undertaken while adhering to the criteria of AA diagnosis as certified through A.I.R.A.S. (Italian Association for Research and Scientific Update; Padua, Italy). Initially, all subjects underwent a blinded examination of both auricles and were evaluated for the presence of angiomas. Identified angiomas were recorded using Romoli’s Sectogram. The blinding comprised not knowing whether the subject would be diagnosed with BC as a first referral, nor whether the subject was a follow-up due to previous BC diagnosis. All subjects with at least one identifiable auricle angioma were selected for further investigation. Baseline demographic data were collected together with convenient data.

After auricle examination, the subjects underwent the outpatient Breast Unit Clinic evaluation and diagnostic pathway (i.e., palpation, ultrasound, mammography, and, if needed, magnetic resonance imaging and biopsy). After completion of breast evaluation and reaching the diagnostic endpoint, the data were analyzed in terms of the presence of auricular angioma and BC diagnosis. Subjects with an identifiable auricle angioma were then allocated to one of two groups based on BC diagnosis. Subjects with a diagnosis of BC were allocated to group K (i.e., the case group), whereas subjects where BC was ruled out were allocated to group NK (i.e., the control group).

**Figure 3:**
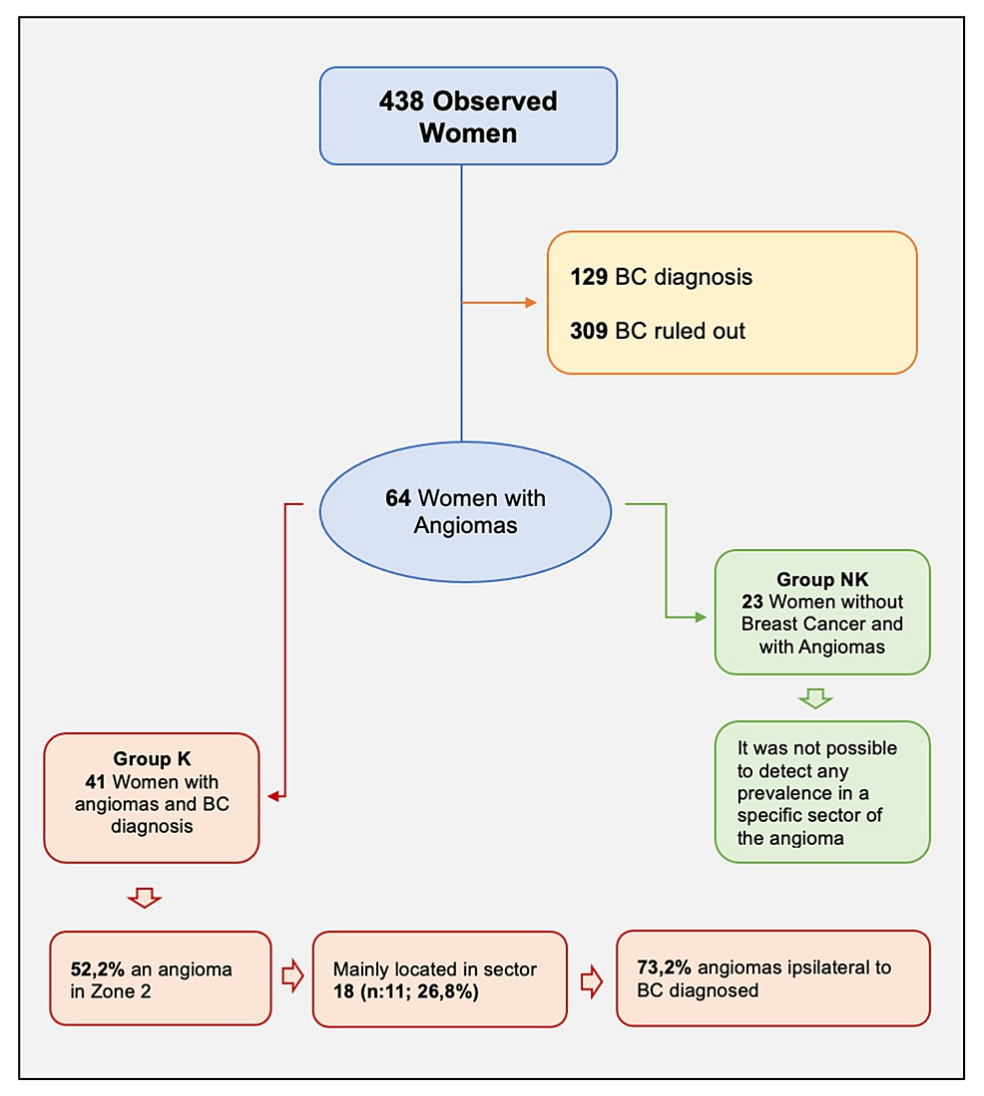
Flow chart of the study BC: breast cancer; K: subjects with auricle angioma and BC; NK: subjects with auricle angioma and BC ruled out

Data collected included age, number of angiomas, Sectogram sector of identified angiomas, laterality of the auricle with the angioma, and laterality of BC. Clinical history of BC was not collected, as the study aimed to correlate the presence of auricular angioma with the presence or absence of BC, and not with the stage of the disease nor with the cancer therapies related to it. A flow chart of the study design is presented in Figure 3.

Data analysis

Descriptive statistics for quantitative variables were reported as means and standard deviations, and qualitative variables were reported as relative frequencies and percentages. Comparisons between patients with and without BC were done using a nonparametric test (i.e., Mann-Whitney U test) for quantitative variables, and odds ratios (ORs) and Fisher's exact test for qualitative variables. Statistical significance was set at 5% (p<0.05). Statistical analyses were performed using STATA18 (StataCorp., College Station, TX, USA).

Ethical aspects

All subjects gave written informed consent to participate in the study. The study protocol was approved by the ethics committee of the scientific association with which the authors are affiliated (protocol number: AIRAS 05012021-CR).

## Results

Evaluation of all subjects

A total of 438 female subjects accessed the outpatient Breast Unit Clinic of Padua and were evaluated for auricle angiomas. The overall prevalence of auricle angiomas within the sample population was 14.6% (i.e., 64 subjects out of 438 evaluated). One hundred and twenty-nine (29%) subjects received a BC diagnosis, whereas BC was ruled out in 309 (71%) subjects. Out of the 129 subjects who had a BC diagnosis, 41 (31%) had an auricle angioma. Out of the 309 subjects where BC was ruled out, 23 (7%) had an auricle angioma. Table 1 describes the number of subjects with or without auricle angiomas and with or without a BC diagnosis. The odds of having an auricle angioma were higher in subjects with a BC diagnosis than in those where BC was ruled out (OR = 5.783; CI 95% =[3.3;10.18]).

**Table 1:** Total subjects evaluated in a contingency table for odds ratio calculation BC: breast cancer. Rows represent the number of subjects with or without angiomas, and columns represent the number of subjects with BC or subjects with BC ruled out, respectively. Also shown are marginal totals and grand totals.

Angioma	BC diagnosis	BC ruled out	Total
Yes	41	23	64
No	88	289	374
Total	129	309	438

Subjects with auricle angioma

A total of 64 subjects (mean age = 60.3 ± 15.0 years) were found to have an identifiable auricle angioma. These 64 subjects were categorized into two groups based on BC diagnosis, for a total of 41 (64.1%) in the case group or group K (i.e., BC diagnosis) and 23 (35.9%) in the control group or group NK (i.e., BC ruled out) (Table 2). A statistically significant difference (p<0.001) was observed between the mean age of subjects in group K (51.6 ± 14.2 years) and those in group NK (65.3 ± 13.3 years).

**Table 2:** Subjects who had an angioma in the auricle SD: standard deviation; K: subjects with auricle angioma and BC; NK: subjects with auricle angioma and BC ruled out; BC: breast cancer

Group	Number	%	Mean age	SD
Total	64	100%	60.3	15
Group K	41	64.1%	51.6	14.2
Group NK	23	35.9%	65.3	13.3

Regarding laterality, of all subjects with an identifiable auricle angioma (n=64), 33 (51.6%) had an auricle angioma on the right auricle, 28 (43.8%) had an auricle angioma on the left auricle, and three (4.7%) had auricle angiomas on both auricles, respectively (Table 3).

**Table 3:** Laterality of auricular angiomas

Auricle	Number	%
Total	63	100%
Right auricle	33	51.6%
Left auricle	28	43.8%
Both auricle	3	4.7%

Of those with an identifiable auricle angioma (n=64), 31 (48.4%) had auricle angiomas located in tumor area II (sectors 15-19), whereas 11 (17.2%) had auricle angiomas located in tumor area I (sectors 4-9), respectively (Table 4).

**Table 4:** Angioma location according to tumor areas I and II

Tumor area and sectors	Number	%
Total	42	100%
Tumor area II (sectors 15-19)	31	48.4%
Tumor area I (sectors 4-9)	11	17.2%

**Figure 4:**
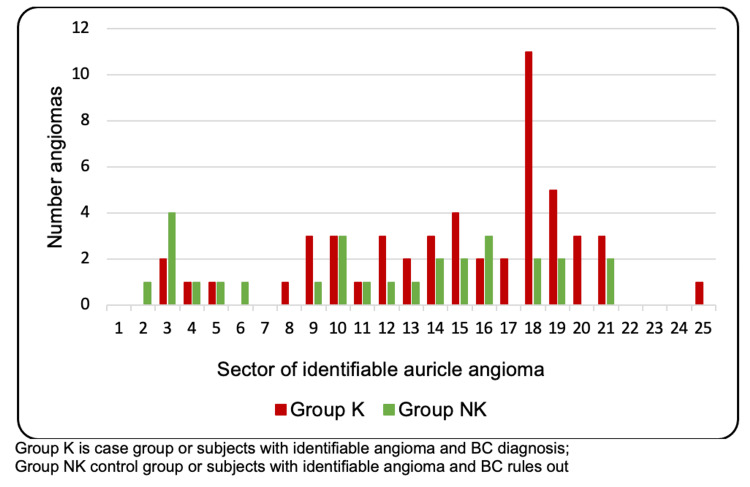
Distribution of angiomas with respect to Romoli sectors The bar graph shows the number of auricle angiomas identified in subjects of group K (red bars) and in subjects of group NK (green bars) by sector of Romoli’s Sectogram. Group K showed a higher number of auricle angiomas in sectors 15-19 (i.e., tumor area II) and in sectors 4-9 (i.e., tumor area I), whereas group NK showed a homogenous distribution. Group K showed the highest number of auricle angiomas in sector 18. Group K: case group; Group NK: control group

In group K (n=41), 21 subjects (51.2%) had an identifiable auricle angioma in tumor area II, as compared to only 10 subjects (43.5%) in group NK (n=23). In group K (n=41), 11 subjects (26.8%) had an identifiable auricle angioma mainly located in sector 18 (i.e., tumor area II), whereas there was no specific sector in group NK with a high prevalence of identifiable auricle angioma (Figure 4). In group K (n=41), 30 subjects (73.2%) had an identifiable auricle angioma on the same side of the BC (p=0.002).

## Discussion

AA diagnosis, as a scientifically validated tool, has been defined in three consecutive steps: inspection, electrical detection (with ESRT), and baresthetic detection (through PPT), after which could take place the needle contact test (NCT), which is a diagnosis test used to select the most efficient points [22]. TCM has always given great importance to inspection. In AA, inspection has been considered the step that must precede all other diagnostic steps. Romoli considered inspection to be the most important AA diagnostic step. According to Romoli and Nguyen, if each diagnostic phase (i.e., inspection, PPT, ESRT, and NCT) was analyzed individually, the one with the highest probability of a correct diagnosis would be inspection [12]. Romoli and Pata have proposed a complete classification of SAs [11], with vascularization alterations being the most common SA in cases of auricles. Vascularization alterations have been found to be the most frequently occurring SAs in acute pathologies (e.g., hyperemia or telagiectasia), whereas others occur more frequently in chronic pathologies (e.g., angiomas) [11]. The French AA School has always given importance to the alignment of AA points. Romoli followed the concept of point alignment in AA diagnosis with his Sectogram [9,23]. To consider the alignment of the SAs on the auricle, the angiomas were recorded by the breast specialist at the Breast Unit Clinic in Padua, using the Sectogram as a method to identify their location. Thus, SAs could be easily identified to be on tumor areas I or II. Other auricle maps that identify tumor areas I and II have not considered SA alignment. Tumor area II has been seen to correspond to sectors 15-19 on Romoli’s Sectogram, which, on international AA maps, correspond to the back, thorax, and breast, as confirmed by different authors [13,14].

Through the simple, quick, and easy observation of auricles of potential BC subjects, auricle angioma was found to be more prevalent in subjects who turned out to have a BC diagnosis than in those who had BC ruled out. Furthermore, the auricle angiomas were predominantly ipsilateral to the side affected by BC, supporting the hypothesis of a correlation consistent with auricle somatotopia. Moreover, auricle angiomas were more localized in sector 18 of Romoli’s Sectogram (i.e., tumor area II) in subjects with BC, as compared to a more homogeneous distribution among those for whom BC was ruled out.

The authors believe that the results of the present study are noteworthy because they corroborate the hypothesis that auricular angiomas found in certain areas in female subjects could be linked to BC pathology, potentially prompting health practitioners to investigate further. In low-income countries where a lack of resources for preventative screening for early BC detection leads to high mortality rates with low incidence rates [17,18], as a simple, low-cost tool, auricle inspection may help health practitioners (especially GPs) to make quick, simple, and cost-effective decisions to refer subjects (especially young women) for BC screening. Furthermore, it is the authors’ opinion that as subjects affected by BC had auricle angiomas mainly localized in tumor area II, it is reasonable to believe that if another type of cancer had been investigated, a different distribution and concentration of auricle angioma would have been found.

Although a statistically significant age difference was found between group K (i.e., auricle angioma and BC diagnosis) and group NK (i.e., auricle angioma and BC ruled out), the authors suspect this phenomenon was due to increased incidence of identifiable auricle angiomas with age. In fact, the presence of identifiable auricle angiomas and the higher number of angiomas mostly in tumor area II in subjects of group K confirm the study’s first (i.e., higher incidence of auricle angiomas in subjects affected by BC) and second (i.e., higher presence of SA identifiable on tumor areas I and II in subjects with BC) hypotheses. Furthermore, the second hypothesis was further confirmed by the fact that identifiable auricle angiomas showed no prevalence in a specific sector in subjects of group NK, confirming the authors’ belief that the higher number of identifiable auricle angiomas may be due to older age.

Study limitations

The main limitation of the study was its small sample size, as a larger sample would further validate our results. Nevertheless, it is the authors’ opinion that the study can be considered easily reproducible for future investigations in different settings, in greater catchment areas, and for different types of cancers, to obtain results that confirm our conclusions. The mechanisms that could explain the higher frequency of auricle angiomas in subjects with BC were not investigated, as the study was limited to describing what was observed through AA diagnosis criteria for the given subjects. The authors refrained from giving opinions or speculating on the mechanisms of auricle angioma origin to avoid cognitive bias. Furthermore, the SAs investigated and the data collected among the sample population were arguably limited due to time and financial constraints.

## Conclusions

An auricle angioma found in tumor areas I or II could be associated with BC on the same side in female subjects. Auricle inspection in AA is a simple, quick, and easy diagnostic tool that does not necessarily require patient compliance. Noticing an SA as easily identifiable as an auricle angioma could help health practitioners (especially GPs) suspect the need for further investigation of a specific pathology, such as BC. Further large-scale studies on the identification of SA, in particular auricle angiomas in Sectogram areas 17-19 in female subjects, would be helpful to further demonstrate the use of auricular inspection to identify potential subjects for referral to BC screening for early detection.

## References

[1] Aoyama N, Fujii O, Yamamoto T (2017). Efficacy of parietal acupoint therapy: scalp acupuncture for neck/shoulder stiffness with related mood disturbance. Med Acupunct.

[2] Hou PW, Hsu HC, Lin YW, Tang NY, Cheng CY, Hsieh CL (2015). The history, mechanism, and clinical application of auricular therapy in traditional Chinese medicine. Evid Based Complement Alternat Med.

[3] Frank ST (1973). Aural sign of coronary-artery disease. N Engl J Med.

[4] Rodríguez-López C, Garlito-Díaz H, Madroñero-Mariscal R, Sánchez-Cervilla PJ, Graciani A, López-Sendón JL, López-de-Sá E (2015). Earlobe crease shapes and cardiovascular events. Am J Cardiol.

[5] Evrengül H, Dursunoğlu D, Kaftan A, Zoghi M, Tanriverdi H, Zungur M, Kiliç M (2004). Bilateral diagonal earlobe crease and coronary artery disease: a significant association. Dermatology.

[6] Oleson TD, Kroening RJ, Bresler DE (1980). An experimental evaluation of auricular diagnosis: the somatotopic mapping or musculoskeletal pain at ear acupuncture points. Pain.

[7] Yan CC, Meng D, Zhang XC, Mao YF, Jia HL, Zhang YC (2021). Discussion on the theory of he-sea point in Neijing (Inner Canon of Yellow Emperor) and Nanjing (Yellow Emperor's Classic of Eighty-one Difficult Issues) [Article in Chinese]. Zhongguo Zhen Jiu.

[8] Nguyen J: L’acupuncture auriculaire avec des aiguilles semi-permanentes soulage la douleur chez les patients cancéreux (2005). Auricular acupuncture with semi-permanent needles relieves pain in cancer patients [Website in French]. Traditionnelle Chinoise.

[9] Romoli M, Mazzoni R (2009). The validation of a new system of transcription of acupuncture points on the ear: the auricular sectogram. Dtsch Z Akupunktur.

[10] Romoli M, Greco F, Giommi A (2016). Auricular acupuncture diagnosis in patients with lumbar hernia. Complement Ther Med.

[11] Romoli M (2015). Diagnosis in Auricular Acupuncture [Book in French]. Casa Editrice Ambrosiana, Milan Italy.

[12] Romoli M, Allais G, Bellu D, De Ramundo B, Gabellari IC, Giommi A, Benedetto C (2010). Ear acupoint detection before and after hysteroscopy: is it possible to clarify the representation of the uterus on the outer ear?. Acupunct Med.

[13] Zhu D, Tian M (1996). Changes of visual findings, electric features and staining of auricles in malignant tumor patients. J Tradit Chin Med.

[14] Huang L-C (1996). Auriculotherapy Diagnosis & Treatment. https://books.google.co.in/books/about/Auriculotherapy_Diagnosis_Treatment.html?id=_6EYcgAACAAJ&redir_esc=y.

[15] Ferlay J, Colombet M, Soerjomataram I, Parkin DM, Piñeros M, Znaor A, Bray F (2021). Cancer statistics for the year 2020: an overview. Int J Cancer.

[16] Arnold M, Morgan E, Rumgay H (2022). Current and future burden of breast cancer: global statistics for 2020 and 2040. Breast.

[17] Francies FZ, Hull R, Khanyile R, Dlamini Z (2020). Breast cancer in low-middle income countries: abnormality in splicing and lack of targeted treatment options. Am J Cancer Res.

[18] Madhav MR, Nayagam SG, Biyani K (2018). Epidemiologic analysis of breast cancer incidence, prevalence, and mortality in India: protocol for a systematic review and meta-analyses. Medicine (Baltimore).

[19] Zielonke N, Kregting LM, Heijnsdijk EA (2021). The potential of breast cancer screening in Europe. Int J Cancer.

[20] Ren W, Chen M, Qiao Y, Zhao F (2022). Global guidelines for breast cancer screening: a systematic review. Breast.

[21] Lovato A (2019). Auricular Acupuncture Theory and Clinic [Book in Italy]. Noi Edizioni, Milan Italy.

[22] Lovato A, Postiglione M, Gagliardi G, Parmagnani M, Biral M, Ceccherelli F (2023). Needle contact test in auricular acupuncture for shoulder pain and where effective auricular acupoints identified are positioned on the map: a controlled study. Eur J Transl Myol.

[23] Lovato A, Ceccherelli F, Gagliardi G, Postiglione M (2022). The medial surface of the auricle: historical and recent maps. What are the possible expectations of the “thumb-index technique”. Medicines (Basel).

